# Censorship in science: How publishing decisions could have shaped the perceived “general consensus” on COVID-19 vaccine safety and efficacy

**DOI:** 10.18632/oncotarget.28828

**Published:** 2026-02-06

**Authors:** Panagis Polykretis, Janci C. Lindsay, Patrizia Gentilini, Nafuko Konishi, Masanori Fukushima

**Affiliations:** ^1^“Allineare Sanità e Salute” Foundation, Milano 20131, Italy; ^2^Independent Medical Scientific Commission (CMSi), Milano 20122, Italy; ^3^Toxicology and Molecular Biology, Toxicology Support Services, LLC., Sealy, TX 77474, USA; ^4^Osaka Metropolitan University School of Medicine, Osaka 545-0051, Japan; ^5^Learning Health Society Institute, Nagoya 450-0003, Japan

**Keywords:** cancer, haematopoietic malignancies, COVID-19, mRNA COVID-19 vaccine

***Commentary on:** Gentilini P, Lindsay JC, Konishi N, Fukushima M, Polykretis P. Exploring the potential link between mRNA COVID-19 vaccinations and cancer: A case report with a review of haematopoietic malignancies with insights into pathogenic mechanisms. Oncotarget. 2026; 16:34–49.* https://doi.org/10.18632/oncotarget.28827.

## INTRODUCTION

It is both a moral and deontological imperative to disclose the publication history of the article “*Exploring the potential link between mRNA COVID-19 vaccinations and cancer: A case report with a review of haematopoietic malignancies with insights into pathogenic mechanisms*”, recently published in *Oncotarget* [1]. Such issue could exemplify a deliberate pattern observed in recent years: the systematic silencing of critical voices challenging the “safe and effective” narrative surrounding mRNA COVID-19 vaccines. By the end of this commentary, it will be evident how a purported “general scientific consensus” may have been artificially engineered by selectively prioritizing studies aligned with the established narrative.

### Submission history

On March 27, 2024, an initial version of the manuscript titled “*A Case Report of Acute Lymphoblastic Leukaemia (ALL)/Lymphoblastic Lymphoma (LBL) Following the Second Dose of Comirnaty*^®^*: An Analysis of the Potential Pathogenic Mechanism Based on Existing Literature*” was posted on Preprints.org [2]. Authored by oncologist Dr. Patrizia Gentilini, molecular biologist Dr. Janci C. Lindsay, physician Dr. Nafuko Konishi, Emeritus Professor of oncology Masanori Fukushima, and structural biologist Dr. Panagis Polykretis, the paper presents a clinical case report of a 39-year-old woman who developed ALL/LBL after her second Comirnaty^®^ dose. It also reviews other cases of heamatological malignancies following mRNA vaccination reported in the literature, along with peer-reviewed evidence on potential mechanisms (including a few preprint studies and FDA/EMA guidelines). As a case report and literature review, the manuscript relies on pre-existing data rather than novel experiments, minimizing risks of design flaws or ambiguous results. Yet, from March 27, 2024, onward, it faced 16 consecutive rejections across journals before final acceptance by *Oncotarget*:

- *Biochemia Medica* - Croatian Society of Medical Biochemistry and Laboratory Medicine (submitted: 30/03/2024; rejected: 01/04/2024);
- *Archives in Endocrinology and Metabolism* - AE&M (submitted: 02/04/2024, rejected: 13/04/2025);
- *Archives of Iranian Medicine* - Academy of Medical Sciences, I.R. Iran (submitted: 16/04/2024, rejected: 24/04/2025);
- *Pathology - Research and Practice* - Elsevier (submitted: 09/10/2024, rejected: 12/10/2024);
- *Annals of Diagnostic Pathology* - Elsevier (submitted: 16/10/2024, rejected: 22/10/2024);
- *Leukemia and Lymphoma* - Taylor and Francis (submitted: 29/10/2024; rejected: 16/11/2024);
- *European Journal of Cancer* - Elsevier (submitted: 29/11/2024; rejected: 04/12/2024 );
- *Critical Reviews in Oncology Hematology* - Elsevier (submitted: 04/12/2024; rejected: 04/12/2024);
- *Oncology Reports* - Spandidos Publications (submitted: 16/12/2024; rejected: 16/12/2024),
- *International Journal of Oncology* - Spandidos Publications (submitted: 20/12/2024; rejected: 20/12/2024);
- *Radiotherapy and Oncology* - Elsevier (submitted: 03/01/2025; rejected: 08/01/2025);
- *Advances in Hematology* - Wiley (submitted: 08/01/2025; rejected: 13/01/2025);
- *Case Reports in Hematology* - Wiley (submitted: 24/01/2025; rejected: 18/04/2025);
- *Case Reports in Oncological Medicine* - Wiley (submitted: 08/05/2025; rejected: 25/09/2025);
- *Current Proteomics* - KeAi Publishing/Elsevier (submitted: 13/05/2025; accepted: 26/08/2025; rejected: 19/09/2025; accepted: 17/10/2025; rejected: 06/11/2025);
- *Oncotarget* - Impact Journals (submitted: 26/11/2025; accepted: 19/01/2026).

Notably, only three submissions (i.e. *Case Reports in Oncological Medicine,* *Current Proteomics* and *Oncotarget*) advanced beyond initial editorial screening to peer-review; the remaining 12 were desk-rejected outright. For *Case Reports in Hematology*, the manuscript lingered for approximately three months without being sent to reviewers (or at least, no peer-review feedback was ever provided), before rejection via a perfunctory note: “*After careful review, we have decided not to publish your manuscript in Case Reports in Hematology*” [3]. In *Case Reports in Oncological Medicine* the manuscript reached peer review, yet after almost 4 months provided only cursory feedback from just one reviewer, who concluded: “*In my opinion, there is not enough evidence to conclude that the administration of Comirnaty*^®^ *can result in ALL*” [4], resulting in rejection.

### The Current Proteomics episode: Twice accepted, then pre-publication rejection

Due to funding constraints for open-access fees, the manuscript was submitted to Current Proteomics, where I served on the Editorial Board, allowing one free publication per year. Submitted on May 13, 2025 (then under Bentham Science, later transitioning to KeAi Publishing, a joint venture co-founded by Elsevier), it underwent three extensive review rounds. An acceptance letter arrived on August 26, 2025, requesting minor formatting changes and template compliance by August 29 [5]. The revised version was submitted on August 28, however publication stalled.

On September 19, 2025, a rejection cited “*concerns about the rationality of the experimental design*”, deeming it insufficient for the conclusions [6]. I rebutted that a case report and review paper inherently lacks an experimental design, warning of resignation and public disclosure. An apology followed on September 20, affirming: “*the core conclusion of your study aligns with rigorous scientific reasoning*”. A fourth revision led to re-acceptance on October 17 by the Co-Editor-in-Chief [7], yet this second acceptance was once again met with prolonged silence from the Editorial Office.

On October 28, after multiple emails to the Editorial Office inquiring about the publication delay, I received a curt but informative message from the Co-Editor-in-Chief: “*Hi, You should contact publisher directly!*” [8]. Why direct me to the Publisher? Was the Publisher blocking publication? And if so, on what scientific grounds could a Publisher intervene between reviewers’ and editors’ decisions? Is the Publisher a subject-matter expert? Escalation to Elsevier’s Research Support connected me to the Journal Manager (Health and Medical Sciences), who cited ongoing “*technical checks*”. After extensive correspondence asking for information, final rejection came on November 6, 2025, from the Co-Editor-in-Chief: “*After in-depth discussions among the Editor-in-Chief, Co-Editor-in-Chief, and Editorial Board, we have made the difficult decision to reject your manuscript... The rejection is based on the fact that the manuscript violates scientific principles and is highly controversial*”, listing nine points [9]. For brevity, only the first two points are discussed here (the rest appear in the cited documentation), as they are indicative of the mindset underlying the remaining seven, to which we were notably denied the opportunity to respond:

1. *“Violates scientific common sense - The development of tumors usually requires changes in genetic material such as gene mutations and chromosomal abnormalities. However, mRNA vaccines only synthesize antigenic proteins in the cytoplasm and do not involve gene integration or replication. COVID-19 mRNA vaccines do not enter the cell nucleus, and thus cannot cause cancer.”*
2. *Linking mRNA vaccines to “insertional mutagenesis” is highly misleading. Insertional mutagenesis is a well-known risk for certain viral vector-based gene therapies that integrate into the host genome. However, mRNA vaccines are non-integrating by their fundamental design, and this risk is considered negligible. Such a false association creates an incorrect and alarming implication.”*

These claims fail to acknowledge the extensive literature on cancer’s multifactorial etiology, focusing narrowly on genetic mutations while overlooking other key drivers. While genetic mutations represent one common pathway, epigenetic alterations, chronic inflammation, immune dysregulation, and tumour microenvironment changes can also initiate oncogenesis without requiring initial DNA mutations [10–15]. Regarding the risk of insertional mutagenesis, the manuscript objectively reports plasmid contamination detected in modRNA genetic vaccines, including Simian Virus 40 (SV40) promoter/enhancer sequences, in the Pfizer/BioNTech pharmaceutical product [16, 17]; the associated risk aligns with FDA and EMA guidelines on residual DNA risk assessment [18, 19]. This represents responsible and objective scientific reporting, rather than misleading speculation. This sequence of events prompted my immediate resignation from the Editorial Board of *Current Proteomics*, as I refuse any association with a journal employing such practices.

All documentation related to the events described (including email exchanges, reviewers’ comments, acceptance and rejection letters, and screenshots of the manuscript’s status over time) has been meticulously preserved by the authors. For the sake of brevity and to maintain focus on the core message, only the most salient elements of this documentation have been included in this article; the complete records remain available to the authors.

In summary, over ~2 years, the manuscript endured 15 submissions and 16 rejections, achieving formal acceptance twice in *Current Proteomics* following peer-review and editorial approval, yet ultimately rejected twice pre-publication. Such post-acceptance reversals represent a profound betrayal of peer-review principles that have formed the bedrock of scientific publishing for over a century. This case raises serious concerns: if scientifically sound dissenting research faces systematic exclusion, the resulting literature becomes selectively curated, artificially constructing “consensus” while marginalizing legitimate scientific discourse. One must question how many other manuscripts are currently enduring similar treatment, potentially never reaching publication, and thus depriving the scientific community of critical information, essential for comprehensive evidence synthesis and patient safety. These ethical and methodological implications demand institutional transparency and reform to preserve the integrity of scientific inquiry.
